# Editorial: Personalized medicine and infectious disease management

**DOI:** 10.3389/fmed.2023.1191147

**Published:** 2023-05-12

**Authors:** Sondes Haddad-Boubaker, Hamdi Mbarek, Hadi M. Yassine

**Affiliations:** ^1^Laboratory of Clinical Virology, WHO Regional Reference Laboratory for Poliomyelitis and Measles for the EMR, Institut Pasteur de Tunis, Tunis, Tunisia; ^2^LR20IPT02, Laboratory of Viruses, Hosts and Vectors, Institut Pasteur de Tunis, The University of Tunis El Manar, Tunis, Tunisia; ^3^Clinical Investigation Center, Institut Pasteur de Tunis, Université Tunis El Manar, Tunis, Tunisia; ^4^Qatar Genome, Qatar Foundation, Doha, Qatar; ^5^Biomedical Research Center, Qatar University, Doha, Qatar

**Keywords:** infectious diseases, genetics, genetic susceptibility, viruses, SNPs

The importance of personalized medicine in the healthcare management of several diseases is well-documented. Still, very little is known about the role of genetics in susceptibility or resistance to infectious diseases (1). Following the emergence of SARS-CoV-2, it became prominent that the genetic background of the patient influences the disease prognosis and treatment. Therefore, multiple genetic databases were established to study precision medicine for COVID-19 (2). This Research Topic gathered different contributions demonstrating the impact of genetic determinants in infectious diseases prognosis and clinical outcome. Ten articles were published in this editorial topic, including five research articles, three reviews, and two case studies.

The first article is titled “*Transient receptor potential vanilloid subtype 1: potential role in infection, susceptibility, symptoms and treatment of COVID-19*” (Liviero et al.). This review article focused on the role of the TRPV-1 channel in the pattern of COVID-19 clinical manifestation, susceptibility, pathogenesis, and therapeutic interventions. TRPV-1 is a receptor involved in immune response, and thus, might be involved in the susceptibility/resistance to SARS-CoV-2 infection. Liviero et al. demonstrated that investigating SNPs of the TRPV-1 gene will raise new therapeutic ways that could help the establishment of effective immune responses resulting in a better clinical outcome.

The other study by Saad et al. focused on the role of ACE 1 in the risk and outcome of SARS-CoV-2 infection. Indeed, the study reported a positive correlation between ACE1 I and the risk of acquiring COVID-19 as well as between the ACE1-D allele and its negative impact following SARS-CoV-2 infection. Thus, the authors suggested that genotyping for ACE1 I/D polymorphism could be useful for better management of the disease. Nevertheless, further evaluation studies are required for validation in different ethnic groups (Saad et al.).

Along the same topic, a study by Ahmed et al. discussed the interaction mechanism of the N501Y mutant recorded in some SARS-CoV-2 variants for ACE2. The authors demonstrated an enhanced affinity of the N501Y mutant S1-RBD with ACE2 compared to the wild phenotype interactions. Such findings might have implications for developing anti-viral drugs against SARS-CoV-2 infection (Ahmed et al.).

On the other hand, Angulo-Aguado et al. investigated the impact of LZTFL1 rs11385942 polymorphism on COVID-19 severity in the Colombian population. They investigated the association of three polymorphisms (ACE rs 4646994, ACE2rs 2285666, and LZTFL1 rs11385942) with COVID-19 short- and long-term outcomes. The study highlighted a positive association between LZTFL1 rs11385942 and COVID-19 severity and the role of nongenetic factors such as clinical signs. They also provided an integrative web-based application as a predictive tool for severity risk assessment. Such tools may be impactful for the management of COVID-19 cases. However, the implementation of this integrative application may pose challenges in areas with limited web accessibility. Further validations for this study are necessary in pre-clinical settings and with a larger cohort to strengthen its findings (Angulo-Aguado et al.).

In a case report study, Schaefer and Bittmann reported on “*Individualized pulsed electromagnetic field therapy in a Long COVID patient using the Adaptive Force (AF) as biomarker*”. This novel diagnostic approach resulted in positive outcomes for one severely affected patient with long COVID-19. They stated that AF reflects the ability of the neuromuscular system to adjust adequately to external powers in an isometric-holding manner. They also reported that the long COVID-19 symptoms did not return after 6 months. Therefore, this case report indicates that this method should be a valuable diagnostic assay for post-COVID-19 illness. Nonetheless, this study was done on only one patient and did not consider genetic polymorphism as a player in response to the treatment (Schaefer and Bittmann).

Immunogenomic is a growing field that combines immunology and genetics to understand how the immune system responds to infection and vaccination. In a review article, Smatti et al. discussed whether host genetics implicate in the response to COVID-19 vaccination, noting that several studies shed light on the contribution of genetic factors in modaling immune responses after vaccination against measles, hepatitis B, rubella, Influenza, and smallpox. In general, genetic variants in genes related to immune response as well as virus replication may shape the individual response to the vaccination. The review highlited the impact of GWAS and other genomic studies to vaccine reponse and adverse understanding. In summary, identifying genetic markers related to the outcome of SARS-CoV-2 infection or response to vaccination may guide healthcare providers in selecting the appropriate treatment, and probably the most reliable vaccine for an individual or an ethnic group (Smatti et al.).

In another comprehensive review, Atallah and Mansour demonstrated the impact of host response-based molecular diagnostics on the clinical management of viral and bacterial infections. They proposed that host-based response diagnostics could be used as a supplement but not a replacement for commonly used pathogen-based diagnostics. Ultimately, accurate and rapid disease diagnosis will be translated into reduced healthcare burden, lesser adverse effects, reduction in the misuse of antibiotics, improvement of public health measures to a better management of infectious diseases and positive patient outcomes (Atallah and Mansour).

Away from acute infections, Chronic Hepatitis B (CHB) continues to be a significant global health challenge due to high morbidity and mortality, in absence of reliable treatments. In their study, Shang et al. investigated the association and clinical relevance of ALDH2 polymorphisms for HBV susceptibility and persistence in a Chinese population. Indeed, it was previously demonstrated ALDH2 contributes in the way of a variety of liver diseases. Genotyping over 1000 participants, they analyzed the role of rs671 and rs1229984 in HBV infection. Compared to healthy controls, rs671-AA genotype frequency was higher in the HBV-infected individuals, especially in the chronic hepatitis B (CHB) group, demonstrating a significant positive association. They also demonstrated that individuals with CHB who harbor the ALDH2 rs671-AA genotype are at higher risk of developing persistent HBV infection and thus, presenting higher HBV load compared with those with GG/GA genotype. These data suggest the possible harmful role of rs671-AA variant in HBV infection, persistence, and chronicity (Shang et al.).

Testing for specific microbes in the central nervous system (CNS) infectious diseases is often tedious and insensitive. Consequently, the delay in identifying the etiological agents and corresponding treatment in patients with CNS infections leads to worse management and outcomes. Chen et al. reported a case study on herpes simplex encephalitis (HSE). In their case, dual mNGS analysis and multiplex PCR (mPCR) were used to identify and semi-quantify the herpes simplex virus (HSV-1). Utilization of combined mNGS and mPCR methods enabled early diagnosis of the infection and disease management using effective treatment. Furthermore, quantifying the viral load along the treatment process can help for better case management (Chen et al.).

Gu et al. reported preliminary findings on the combined effect of low-dose trimethoprim-sulfamethoxazole (TMP/SMX) and clindamycin on severe pneumocystis pneumonia (PCP) following renal transplantation. Including 20 patients in their study, the authors claimed that the use of this combined treatment on PCP patients was more effective than the single use of TMP/SMX alone. They also demonstrated the safety of such treatment, especially in patients that are intolerant to the standard dose of TMP/SMX. However, Further molecular investigation was required to confirm the improved patient outcome (Gu et al.).

Finally, personalized or precision medicine is a growing approach to improve patient care by applying the right intervention at the right time. According to the GWAS Catalog statistics (OCT 2020), out of 4,761 publications, only eighty-six were related to infectious diseases (ID) (1.8%). Further, only 2,496 associations were ID-related (1.1%) out of 213,519 total associations. With the emergence of SARS-CoV2, most studies have been focused on COVID-19, which was also reflected in this special topic. However, with the significant progress and achievements in this field, we anticipate that other ID, particularly those linked to complex diseases like cancer and neurodegenerative conditions, will be investigated.

The ultimate aim of this Research Topic was to shed light on the importance of genetics and personalized medicine in improving ID management and treatment. Several topics were discussed to highlight the importance of genetic testing in understanding disease susceptibility, prognosis, treatment, as well as drug and vaccine utilization.

## Author contributions

SH-B and HY wrote the initial draft. All authors reviewed and approved the last version.

## References

[1] Zeberg H Paabo S. The major genetic risk factor for severe COVID-19 is inherited from Neanderthals. Nature. (2020) 587:610–2. 10.1038/s41586-020-2818-3 32998156

[2] Russo R Andolfo I Lasorsa VA Iolascon A Capasso M. Genetic analysis of the coronavirus SARS-CoV-2 host protease TMPRSS2 in different populations. Front Genet. (2020) 11:872. 10.3389/fgene.2020.00872 32849840 PMC7417663

